# Production of low-calorie biscuits using stevia extract and dietary fibers from oats and fenugreek for functional food applications

**DOI:** 10.1038/s41538-026-00768-w

**Published:** 2026-03-23

**Authors:** Mohamed Ahmed Hassan, Mustafa Abdelmoneim Mustafa, Asem M. Abdelshafy, Walaa Kobacy

**Affiliations:** 1 Department of Food Science and Technology, Faculty of Agriculture, Al-Azhar University—Assiut Branch, Assiut, Egypt; 2 Department of Food Science and Nutrition, Faculty of Agriculture, Sohag University, Sohag, Egypt

**Keywords:** Biochemistry, Biotechnology, Health care, Plant sciences

## Abstract

Biscuits are a popular, low-cost, and ready-to-eat food. However, high sugar level and low fiber content of biscuits are linked to serious health risks such as diabetes and obesity. The objective of this study was to develop low-calorie biscuits with enhanced dietary fiber content by incorporating stevia extract alongside oat and fenugreek fibers. Five distinct formulations were developed using white flour and other ingredients. These were designated as control (100% refined wheat flour with 30 g sugar), WG-3 (100% refined wheat flour with 3 g galactomannan and 27 mL stevia extract), WG-6 (100% refined wheat flour with 6 g galactomannan and 27 mL stevia extract), WB-3 (100% refined wheat flour with 3 g *β*-glucan and 27 mL stevia extract), and WB-6 (100% refined wheat flour with 6 g *β*-glucan and 27 mL stevia extract). The study found that substituting sugar with a combination of stevia extract and dietary fibers resulted in a reduction of both non-reducing sugars and caloric level. Also, the substitution of sugar with stevia extract and dietary fibers boosted antioxidant activity and suppressed α-amylase and α-glucosidase enzymes. Among all formulations, WB-6 demonstrated superior bioactivity, achieving an antioxidant activity of 8.63% and inhibition rates of 33.87% against α-amylase and 42.53% against α-glucosidase. The study found that biscuits made with stevia extract and dietary fibers are a promising low-calorie, high-fiber functional food.

## Introduction

Biscuits are very popular ready-to-eat foods due to their long shelf-life and affordable cost. They have been a convenient food for centuries, sustained by their versatility and adaptability^1^. The demand for biscuits grew in several countries during the COVID-19 lockdowns, as their shelf-stable nature was highly appreciated when food purchases became less frequent^2^. Its primary ingredients are flour, fat, sugar, water, milk, salt, and chemical leavening. Due to their highly adaptable recipe, biscuits are an ideal medium for incorporating nutrients and functional ingredients^3^.

However, the high sugar level in biscuits is associated with numerous adverse health outcomes, including an increased risk of type II diabetes, obesity, dental caries, and coronary heart disease^4^. Furthermore, a key nutritional limitation of biscuits is their low fiber content, which diminishes their health-promoting properties^5^. In response to these concerns, many strategies such as the reduction of sugar content and the incorporation of healthier ingredients are being explored^6^.

With a sweetness potency estimated at 200–400 times that of sucrose, stevia-derived sweeteners deliver a non-caloric sweetening option, suitable for both the general public and specific populations like diabetics seeking to reduce caloric and sugar consumption. It is used in many foods and drinks, and though it can have a slight aftertaste, manufacturers have found ways to mask it^7^. Stevioside is a natural sweetener extracted from Stevia leaves. It is classified as a diterpene glycoside, meaning its structure is built from a steviol core and three glucose molecules^8^. It is characterized as a white powder with high aqueous solubility, it demonstrates excellent stability during storage and processing^9^.

On the other hand, soluble fibers such as *β*-glucans are highly recommended for diabetics due to their ability to regulate blood sugar, improve cholesterol levels, aid in weight management, and provide other metabolic benefits^10^. It is a non-starch polysaccharide composed of D-glucose monomers linked by *β*-glycosidic bonds. In cereals such as barley and oats, *β*-glucans consists of linear β-(1 → 4)-linked D-glucopyranosyl units interrupted by single β-(1 → 3) linkages at irregular intervals, forming mixed-linkage (1 → 3), (1 → 4)-β-D-glucan^11^.

Moreover, galactomannan is a natural polysaccharide chiefly found in the endosperm of some plant seeds such as fenugreek^12^. Due to its high molecular weight and non-ionic nature, galactomannan forms stable, viscous solutions in water^13^. Its chemical structure consists of a β-(1–4)-D-mannan backbone with single α-(1–6)-D-galactose branches^12^. Dietary fiber contributes significantly to diabetes management through its ability to slow glucose absorption. This mechanism assists in the control of overall blood glucose levels and the attenuation of postprandial glucose excursions^14^.

**Fig. 1:**
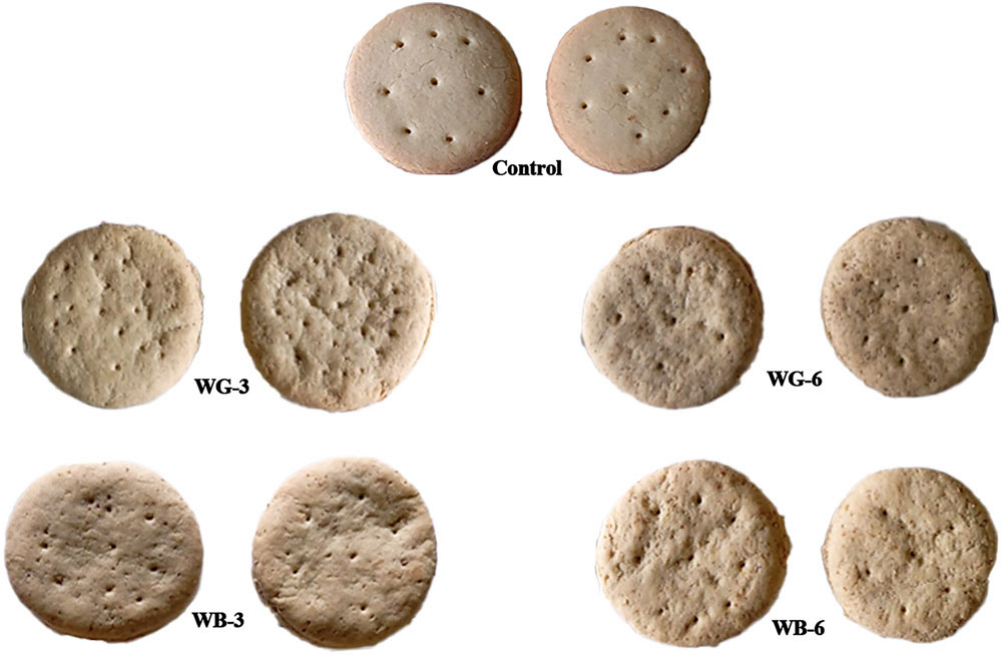
The prepared biscuits. **Control** (100% refined wheat flour), **WG-3** (100% refined wheat flour with 3 g galactomannan and stevia extract), **WG-6** (100% refined wheat flour with 6 g galactomannan and stevia extract), **WB-3** (100% refined wheat flour with 3 g *β*-glucan and stevia extract) **WB-6** (100% refined wheat flour with 6 g *β*-glucan and stevia extract).

Some studies were conducted on the production of low-calorie biscuits using stevia extract^15,16^. Research has also investigated high-fiber biscuits incorporating dietary fiber from natural sources, such as oat *β*-glucan^17^. However, there is a lack of studies on the application of stevia extract combined with specific dietary fibers in formulating functional biscuits. A novel aspect of this research is the formulation of functional biscuits using stevia extract in combination with dietary fibers (*β*-glucan from oats or galactomannan from fenugreek) to achieve low-calorie and high-fiber content (Fig. 1). The effect of adding stevia extract and dietary fibers on dough rheological behavior and functional properties of biscuit formulations were investigated. Moreover, the chemical composition and antioxidant activity of produced biscuits were evaluated. Also, the enzyme inhibitory activities of α‑amylase and α‑glucosidase of prepared biscuits were determined.

## Results and discussions

### Impact of stevia extract, galactomannan, and *β*-glucan on flour formulations and dough characteristics

#### Functional properties of flour formulations

The functional properties of flour formulations including water holding capacity (WHC), oil holding capacity (OHC) and solubility are illustrated in Table 1. WHC describes how well proteins and other food components can bind and retain water. This property is fundamental in determining key qualities such as texture, tenderness, and structural integrity in many food products^18,19^. Multiple variables impact the hydration properties of foods, notably the composition of amino acids, surface polarity, hydrophobicity, pH and temperature^20,21^. The WG-6 treatment exhibited the highest value of WHC (165.4%) followed by WG-3 which had 131.4% WHC.

**Table 1:** The functional properties of flour formulations, and color properties of biscuits

Functional properties of flour formulations				Color properties of biscuits		
**Treat**	**WHC** (%)	**OHC** (%)	**Solubility** (%)	L*	a*	b*
**Control**	77.90 ± 1.70^c^	89.68 ± 1.07^a^	14.76 ± 0.36^a^	78.72 ± 0.23^a^	9.87 ± 0.15^a^	23.91 ± 0.36^a^
**WG-3**	131.4 ± 2.80^b^	87.11 ± 2.19^b^	13.62 ± 0.30^b^	71.95 ± 0.71^c^	7.06 ± 0.09 ^d^	22.05 ± 0.45^b^
**WG-6**	165.4 ± 7.12^a^	80.75 ± 1.15^c^	13.76 ± 0.76^b^	70.51 ± 1.21 ^d^	6.91 ± 0.12 ^d^	21.91 ± 0.50^b^
**WB-3**	82.30 ± 5.50^c^	85.25 ± 1.06^b^	14.92 ± 0.30^a^	74.51 ± 0.69^b^	7.90 ± 0.03^b^	23.74 ± 0.23 ^a^
**WB-6**	88.90 ± 3.56^c^	82.07 ± 0.44^c^	15.06 ± 0.54^a^	74.16 ± 0.16^b^	7.54 ± 0.01^c^	22.49 ± 0.07^b^

Control (100% refined wheat flour), WG-3 (100% refined wheat flour with 3 g galactomannan and stevia extract), WG-6 (100% refined wheat flour with 6 g galactomannan and stevia extract), WB-3 (100% refined wheat flour with 3 g *β*-glucan and stevia extract) WB-6 (100% refined wheat flour with 6 g *β*-glucan and stevia extract), WHC (Water Holding Capacity), OHC (Oil Holding Capacity). L* (lightness), a* (redness), b* (yellowness). Values presented as the means of triplicate ± standard deviation. Means within a column with different superscript small letters are significantly different (p ≤ 0.05).

Moreover, the WB-6 and WB-3 treatments showed WHC values of 88.90% and 82.30%, respectively, while control sample showed WHC value of 77.90%. The increase of WHC in samples containing galactomannan and *β*-glucan may be due to the increase in dietary fiber content. These results are in line with those obtained by Mert^22^, who indicated that the dietary fiber content improved the functional properties of food products, including antioxidant activity.

OHC is the ability of ingredients like proteins to absorb and retain oil, which is vital for enhancing the texture, flavor, and stability of food products^23^. Particle size and surface area are key determinants of the oil absorption capacity in food powders and flours^24^. The results showed that the OHC of samples containing galactomannan or *β*-glucan were decreased whereas, the control sample recorded the highest value (89.68%). The degradation of lipids during the kneading may be responsible for the decrease in OHC of biscuits^25^. Furthermore, solubility is related to soluble substances such as sugar, amylose, and protein in the food. WB-6 exhibited the highest solubility value (15.06%) followed by WB-3 (14.90%). Moreover, the flour formulation containing *β*-glucan at two levels exhibited lower solubility value than control sample (14.76%). The decrease in solubility may result from the soluble substances being degraded, or complexing with insoluble substances^26^.

#### Rheological properties of prepared dough

**Fig. 2:**
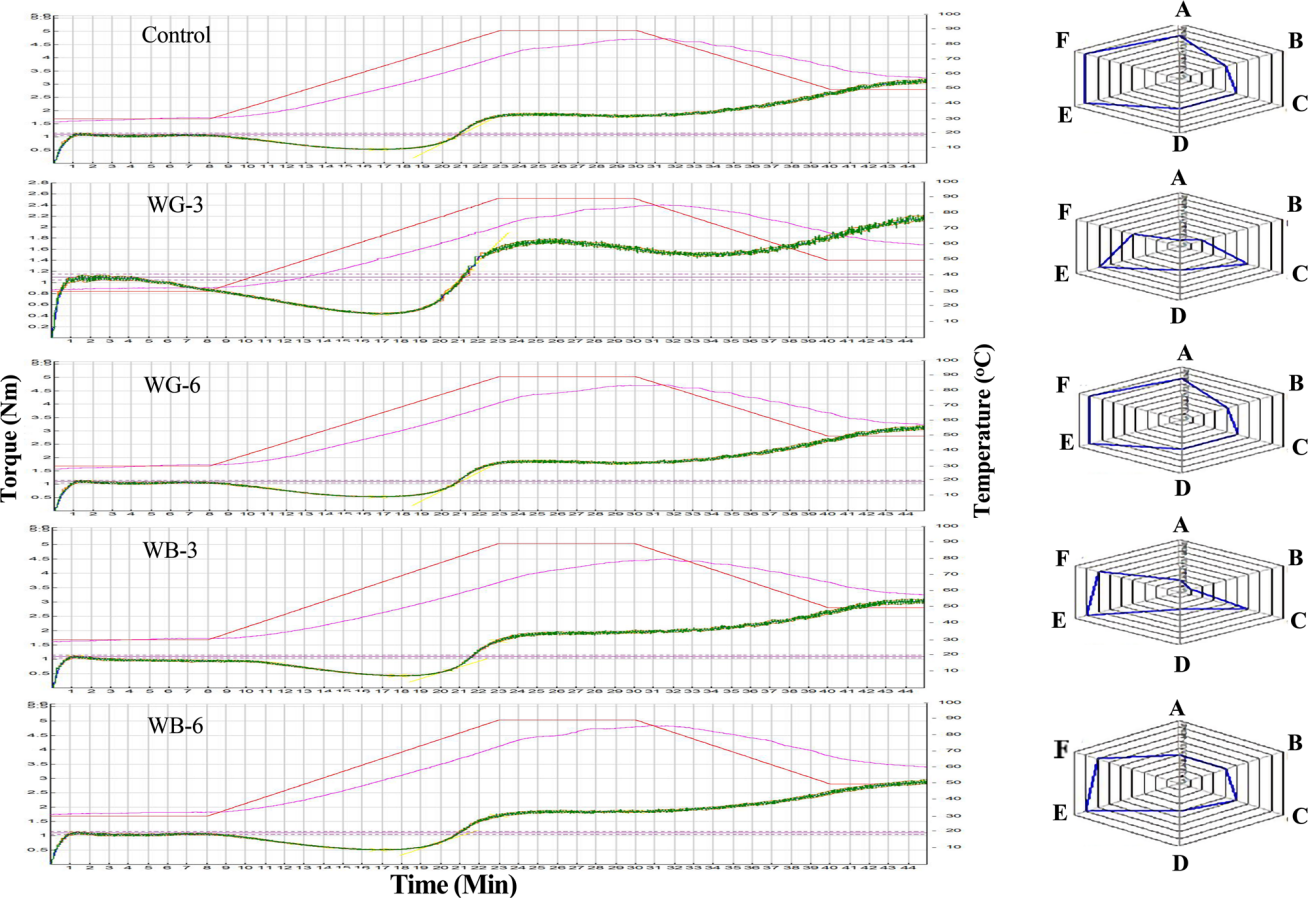
The rheological properties of formulations dough using Mixolab. **Control** (100% refined wheat flour), **WG-3** (100% refined wheat flour with 3 g galactomannan and stevia extract), **WG-6** (100% refined wheat flour with 6 g galactomannan and stevia extract), **WB-3** (100% refined wheat flour with 3 g *β*-glucan and stevia extract) **WB-6** (100% refined wheat flour with 6 g *β*-glucan and stevia extract). **A** absorption, **B** mixing, **C** gluten + , **D** viscosity, **E** amylase, **F** retrogradation.

The effects of stevia leaves extract, fenugreek galactomannan (3% and 6%) and oat *β*-glucan (3% and 6%) on the thermomechanical properties of formulations dough were presented in Table 2 and Fig. 2. The inclusion of both galactomannan and *β*-glucan increased the water absorption of wheat flour. The water absorption increased from 53.2% for control treatment to 57.2%, 56.9%, 55.3%, and 58.60% for WG-3, WG-6, WB-3, and WB-6, respectively. The fortification of wheat flour with *β*-glucan, sourced from various origins, has been widely reported to result in elevated water absorption^27,28^. The molecular structure of hydrocolloids and dietary fiber, characterized by a high density of hydroxyl groups, promotes significant (p ≤ 0.05) water absorption via the formation of hydrogen bonds^29^.

**Table 2:** Effects of incorporation galactomannan and *β*-glucan with wheat flour on rheological properties of dough

Parameters	Control	WG-3%	WG-6%	WB-3%	WB-6%
**WA (%)**	53.2 ± 0.035 ^d^	57.2 ± 0.23^b^	56.9 ± 0.09^b^	55.3 ± 0.43^c^	58.6 ± 0.59^a^
**DDT (min)**	1.53 ± 0.057^b^	2.52 ± 0.017^a^	1.53 ± 0.041^b^	1.18 ± 0.012 ^d^	1.35 ± 0.014^c^
**Stability (min)**	7.23 ± 0.11 ^d^	6.27 ± 0.021^e^	8.43 ± 0.08^b^	7.62 ± 0.023^c^	8.89 ± 0.12^a^
**Amplitude**	0.07 ± 0.001^a^	0.08 ± 0.001^a^	0.08 ± 0.000^a^	0.10 ± 0.005^a^	0.09 ± 0.005^a^
**C1 (Nm)**	1.10 ± 0.012^a^	1.09 ± 0.052^a^	1.10 ± 0.037^a^	1.09 ± 0.015^a^	1.10 ± 0.006^a^
**C2 (Nm)**	0.53 ± 0.008^b^	0.43 ± 0.003^c^	0.58 ± 0.006^a^	0.43 ± 0.004^c^	0.54 ± 0.01^b^
**C3 (Nm)**	1.86 ± 0.018^a^	1.75 ± 0.011^b^	1.91 ± 0.010^a^	1.63 ± 0.013^c^	1.71 ± 0.002^b^
**C4 (Nm)**	1.78 ± 0.006^b^	1.52 ± 0.002 ^d^	1.63 ± 0.012^c^	1.95 ± 0.024^a^	1.83 ± 0.009^b^
**C5 (Nm)**	3.12 ± 0.023^a^	2.19 ± 0.009^c^	3.10 ± 0.028^a^	3.04 ± 0.019^a^	2.81 ± 0.011^b^
**RET %**	42.94	30.59	47.24	35.85	34.87

WA (Water absorption), DDT (dough development time), RET (Retrogradation), C1: used to calculate water absorption, C2: is a measure of dough weakening, C3: is an indication of starch gelatinization during the heating and cooking, C4: indication about the rate of enzymatic hydrolysis and the stability of the hot gel formed and C5: an indication of retrogradation or re-ordering of starch molecules during the cooling. Control (100% refined wheat flour), WG-3 (100% refined wheat flour with 3 g galactomannan and stevia extract), WG-6 (100% refined wheat flour with 6 g galactomannan and stevia extract), WB-3 (100% refined wheat flour with 3 g *β*-glucan and stevia extract) WB-6 (100% refined wheat flour with 6 g *β*-glucan and stevia extract). Values presented as the means of triplicate ± standard deviation. Means within a column with different superscript small letters are significantly different (*p* ≤ 0.05).

The dough development time (DDT) is the maximum time to reach C1 torque and it was 1.53 min for control and WG-6 samples whereas it reduced to 1.18 min and 1.35 min in WB-3 and WB-6 samples, respectively (Table 2). WG-3 was the only treatment that resulted in an increase in DDT (2.52 min). Mohebbi et al.^27^ reported that the supplementation with 0.8–1.2% *β*-glucan significantly (*p* ≤ 0.05) decreased the DDT. These effects were primarily due to dietary fiber interfering with gluten network formation, a consequence of gluten dilution^30^. However, Wang et al.^31^ found that incorporating pea fiber into wheat dough had no statistically significant differences (*p* > 0.05) on its development time.

In most cases, the stability of dough was significantly (*p* ≤ 0.05) greater with the addition of *β*-glucan and galactomannan Fig. 2. WB-6 showed the highest value of stability (8.89 min) compared to the control sample (7.23 min). This could be explained by higher interactions between *β*-glucan or galactomannan, water, and flour proteins (gluten). The resulting dough was firmer and more stable during mixing. These results are in line with those obtained by Blibech et al.^32^, who found that the addition of galactomannan (2%) extracted from Tunisian carob seed significantly (*p* ≤ 0.05) increased the dough stability. Amplitude is the curve width at C1 and indicates protein quality or dough elasticity^33^. Amplitude values of dough containing both galactomannan and *β*-glucan had no significant differences (p > 0.05) compared to control (0.07 Nm) and ranged between 0.08 and 0.10 Nm.

The addition of galactomannan and *β*-glucan did not significantly (*p* > 0.05) increase C1, which is the maximum consistency reached during mixing. The control sample had a C1 value of 1.10 Nm. However, according to Skendi et al.^34^ the C1 value increased during dough formation when barley *β*-glucan was added to wheat flour. These different results may be due to the type and molecular weight of *β*-glucan as well as the combination of *β*-glucan with stevia extract. A decrease in dough consistency (C2 value) is induced by the protein unfolding and destabilization caused by the combined effects of heating and shear stress^35^. The degree to which the dough softens indicates the quality and strength of its protein. Additionally, C2 represents the torque measured at the onset of starch gelatinization. The highest C2 value (0.58 Nm) was recorded in WG-6, followed by WB-6 (0.54 Nm) and control (0.53 Nm), while the lowest values were observed in WG-3 and WB-3. The presence of higher amount of insoluble *β*-glucan or galactomannan may be the main reason for this result. An increase in C2, indicating a delay in protein weakening, was observed following the addition of pea fiber to wheat flour^36^.

The addition of both galactomannan and *β*-glucan to wheat flour gave a lower peak viscosity (C3), it was 1.86 Nm for control, whereas it decreased to 1.75 Nm, 1.63 Nm and 1.71 Nm in WG-3, WB-3, and WB-6, respectively. This could be explained by the competition of water between starch and galactomannan or *β*-glucan^32^. The highest C3 value (1.91 Nm) was recorded for WG-6. The elevated torque observed in dough containing galactomannan may be attributed to increased viscosity or consistency, resulting from the hydration of the galactomannan. Additionally, hydrocolloids can immobilize water molecules, which leads to an increase in the effective starch concentration^35^.

The parameter C4 represents the minimum torque, reflecting a decrease in hot paste consistency due to shear thinning and the breakdown of swollen starch granules. Dough containing *β*-glucan (WB-3) exhibited significant (*p* ≤ 0.05) higher C4 value (1.95 Nm) compared to the control sample which recorded C4 value of 1.78 Nm. This indicates that β-glucan helps reduce paste breakdown, contributing to greater stability. In samples containing both galactomannan and *β*-glucan, values ranged from 1.52 Nm to 1.95 Nm. This lower breakdown is likely attributed to *β*-glucan, which acts as a physical barrier due to its dense structure, impeding the shear thinning of the hot starch paste. Furthermore, *β*-glucan also hinders alpha-amylase activity, thereby reducing starch liquefaction and shear thinning following gelatinization^37^. Gavilighi et al.^38^ observed a reduction in starch breakdown with the addition of 5% *β*-glucan. In contrast, C4 values in WG-3 and WG-6 were significantly (p ≤ 0.05) lower than those in the control sample. The observed decrease may be a consequence of the low molecular weight of fenugreek galactomannan^32^.

The slight decrease in the C5 value observed upon adding galactomannan and *β*-glucan to wheat flour indicates a reduction in starch retrogradation. This stage involves starch retrogradation, the process by which starch molecules reassociate. A lower setback value is indicative of a decelerated staling process. The percent increase in torque from C4 to C5 (C5-C4%), representing the extent of retrogradation, was 42.94% in the control dough. In contrast, doughs containing 3% galactomannan and 3–6% *β*-glucan showed a lower range of 30.59% to 35.85%, indicating reduced retrogradation due to the presence of non-starch polysaccharides. *β*-glucan impedes the retrogradation of starch, a key mechanism of staling, through its hygroscopic properties^34^. Furthermore, the adding locust bean galactomannan (2%) led to decreasing C5 from 2.3 (Nm) for control bread to 1.9 (Nm) for bread supplemented with galactomannan^32^.

### Impact of stevia extract, galactomannan, and *β*-glucan on biscuit characteristics

#### Chemical composition of biscuits

The chemical composition of biscuits prepared from wheat flour, dietary fibers, and stevia extract was illustrated in Table 3. Compared to control sample, the addition of stevia extract significantly (*p* ≤ 0.05) improved the protein content of all treated biscuits. On the other hand, no significant differences (*p* > 0.05) were observed among treated biscuits with stevia extract. The WG-3, WG-6, WB-3, and WB-6 biscuits showed protein contents ranging from 8.08% to 8.25% (On dry basis) compared to control sample (7.52%). Moreover, adding galactomannan and *β*-glucan at a level of 6% significantly (*p* ≤ 0.05) increased the dietary fiber content of biscuits from 0.28% (for control sample) to 0.67% and 0.69%, respectively. No significant differences (*p* > 0.05) were observed in the dietary fiber content between biscuits containing galactomannan or *β*-glucan at a level of 3% and control sample.

**Table 3:** Gross Chemical composition of biscuits

Parameters (%)	Control	WG-3	WG-6	WB-3	WB-6
**Moisture**	2.49 ± 0.55^e^	2.76 ± 0.30^c^	4.13 ± 0.42^a^	2.78 ± 0.66^c^	3.24 ± 0.24^b^
**Protein**	7.52 ± 0.23^b^	8.25 ± 0.16^a^	8.16 ± 0.14^a^	8.14 ± 0.10^a^	8.08 ± 0.27^a^
**Fiber**	0.28 ± 0.06^b^	0.36 ± 0.08^b^	0.67 ± 0.07^a^	0.37 ± 0.05^b^	0.69 ± 0.02^a^
**Ash**	1.29 ± 0.05 ^d^	1.86 ± 0.10^ab^	1.64 ± 0.09^c^	1.94 ± 0.04^a^	1.73 ± 0.08^bc^
**Fat**	14.87 ± 0.08^a^	15.06 ± 0.36^a^	14.92 ± 0.30^a^	15.13 ± 0.26^a^	15.03 ± 0.12^a^
Carbohydrates*	73.55 ± 0.32^a^	71.71 ± 0.49^b^	70.48 ± 0.31^c^	71.64 ± 0.40^b^	71.23 ± 0.29^b^
**Reducing sugar**	0.43 ± 0.17^ab^	0.45 ± 0.001^a^	0.40 ± 0.029^bc^	0.43± 000^ab^	0.38 ± 0.02^c^
**Non-reducing sugar**	12.34 ± 0.63^a^	1.14 ± 0.020^b^	1.08 ± 0.031^b^	1.16 ± 0.10^b^	1.03 ± 0.09^b^

Control (100% refined wheat flour), WG-3 (100% refined wheat flour with 3 g galactomannan and stevia extract), WG-6 (100% refined wheat flour with 6 g galactomannan and stevia extract), WB-3 (100% refined wheat flour with 3 g *β*-glucan and stevia extract) WB-6 (100% refined wheat flour with 6 g *β*-glucan and stevia extract). Values presented as the means of triplicate ± standard deviation. Means within a column with different superscript small letters are significantly different (*p* ≤ 0.05).

*Carbohydrates by deference.

Also, the addition of stevia extract significantly (*p* ≤ 0.05) improved the ash content of biscuits. The highest ash content (1.94%) was observed in the WB-3 biscuits, followed by the WG-3 biscuits (1.86). In contrast, the control sample recorded the lowest value of ash content (1.29%). The total carbohydrates showed significant decrease (*p* ≤ 0.05) in all treated biscuits compared to control biscuits. Results indicated that substituting sugar with stevia extract and dietary fibers resulted in significant reduction (*p* ≤ 0.05) in non-reducing sugar from 12.34% (for control) to values ranged from 1.03 to 1.16% for stevia-treated biscuits. No significant differences (*p* > 0.05) in the fat contents between treated biscuits and control biscuits. The high protein and ash contents in the biscuits may be attributable to the significant proportion of protein and ash found in the stevia extract^39,40^.

#### Calorie assessment of prepared biscuits

**Fig. 3:**
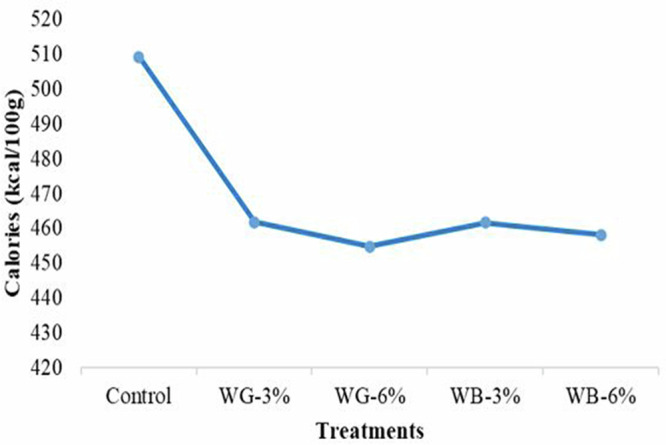
Calorie assessment of biscuit samples. **Control** (100% refined wheat flour), **WG-3** (100% refined wheat flour with 3 g galactomannan and stevia extract), **WG-6** (100% refined wheat flour with 6 g galactomannan and stevia extract), **WB-3** (100% refined wheat flour with 3 g *β*-glucan and stevia extract) **WB-6** (100% refined wheat flour with 6 g *β*-glucan and stevia extract).

Figure 3 demonstrates the caloric variation in biscuits resulting from the substitution of sugar with stevia extract and the inclusion of dietary fibers. The analysis revealed that the inclusion of stevia extract and dietary fibers led to a significant lowering (*p* ≤ 0.05) of the calorie content in biscuit samples. Stevia-treated biscuits (WG-3, WG-6, WB-3, and WB-6) exhibited lower calorie values, ranging from 454.76 kcal/100 g to 461.74 kcal/100 g, compared to the control sample, which had the highest value of 509.19 kcal/100 g. No significant differences (*p* > 0.05) were observed among stevia-treated biscuits. Results indicated that completely substituting sugar with stevia extract and dietary fibers led to an 11% reduction in calories. This decrease is attributed to the elimination of sugar and the use of the zero-calorie sweetener, stevioside. Our results align with those of Vatankhah et al.^41^ who reported a 15% reduction in the caloric value of biscuits following the complete substitution of sugar with stevioside.

#### Physical properties of biscuits

The mean values of physical characteristics of biscuits, including diameter, thickness, spread ratio and spread factor are shown in Table 4. The diameter of biscuit samples containing galactomannan and *β*-glucan were significantly decreased (*p* ≤ 0.05) compared to control which recorded 4.66 ± 0.08, and WG-6 biscuits recorded the lowest diameter 3.93 ± 0.16 cm. The observed decrease in diameter can be attributed to an inability of the biscuit’s cellular matrix to effectively retain gases generated during the proofing and baking stages^42^. Moreover, data recorded increments of thickness from 0.53 ± 0.02 for the control sample to 0.79 ± 0.07, 0.84 ± 0.04, 0.91 ± 0.04, and 0.98 ± 0.06 cm for WG-3, WG-6, WB-3, and WB-6 biscuits, respectively. Moreover, the spread ratio and spread factor of biscuit samples containing galactomannan and *β*-glucan were decreased compared to control which recorded 8.87 ± 0.36 and 100.00 ± 0.00, respectively. The WB-6 biscuits recorded the lowest spread ratio and spread factor values by 4.21 ± 0.32 and 47.51 ± 0.49, respectively. The reduction of sugar increased water absorption by gluten, leading to a more elastic dough. This elasticity caused the dough to thicken and resist spreading during rolling^43^. A potential mechanism for the reduced diameter and increased thickness could be the lowered dough viscosity caused by removing sugar from the formulation^44^.

**Table 4:** Physical properties of biscuits

Treatments	Diameter (cm)	Thickness (cm)	Spread ratio (%)	Spread factor
**Control**	4.66 ± 0.08^a^	0.53 ± 0.02 ^d^	8.87 ± 0.36^a^	100.00 ± 00^a^
**WG-3**	3.99 ± 0.12^c^	0.79 ± 0.07^c^	5.07 ± 0.36^b^	57.18 ± 0.74^b^
**WG-6**	3.93 ± 0.16^c^	0.84 ± 0.04^c^	4.71 ± 0.28^c^	53.12 ± 0.26^c^
**WB-3**	4.13 ± 0.05^b^	0.91 ± 0.04^b^	4.52 ± 0.17 ^cd^	50.98 ± 0.41 ^d^
**WB-6**	4.11 ± 0.09^b^	0.98 ± 0.06^a^	4.21 ± 0.32 ^d^	47.51 ± 0.49^e^

Control (100% refined wheat flour), WG-3 (100% refined wheat flour with 3 g galactomannan and stevia extract), WG-6 (100% refined wheat flour with 6 g galactomannan and stevia extract), WB-3 (100% refined wheat flour with 3 g *β*-glucan and stevia extract) WB-6 (100% refined wheat flour with 6 g *β*-glucan and stevia extract). Values presented as the means of triplicate ± standard deviation. Means within a column with different superscript small letters are significantly different (*p* ≤ 0.05).

#### Color properties of biscuits

The color values (L*, a*, and b*) of prepared biscuit samples are displayed in Table 1. L*, a*, and b* values recorded 78.72 ± 0.23, 9.87 ± 0.15, and 23.91 ± 0.36 for control biscuits. Among biscuits treated with stevia and dietary fiber, WB-3 biscuits showed the highest L*, a*, and b* values (74.51 ± 0.69, 7.90 ± 0.03, and 23.74 ± 0.23, respectively) followed by WB-6 (74.16 ± 0.16, 7.54 ± 0.01, and 22.49 ± 0.07, respectively). It was observed that there was a decrease in lightness, redness, and yellowness in all biscuits treated with stevia compared to control biscuits. The presence of pigments, specifically chlorophylls A and B and carotenoids, is the principal cause of the dark coloration in stevia extract, which often has an undesirable effect on the color of final food products^40^. These findings align with those of Sulaiman et al.^40^, who also observed a reduction in lightness when stevia extract was added to low-calorie cake.

#### Antioxidant activity of biscuits

**Fig. 4:**
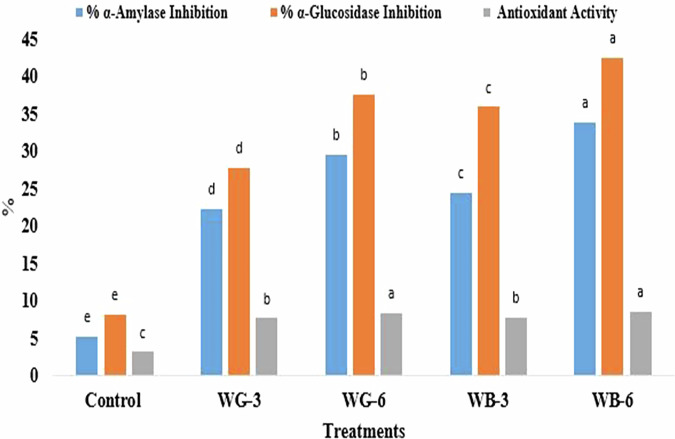
α-amylase and α‑glucosidase inhibitory effects and antioxidant activity of biscuits. **Control** (100% refined wheat flour), **WG-3** (100% refined wheat flour with 3 g galactomannan and stevia extract), **WG-6** (100% refined wheat flour with 6 g galactomannan and stevia extract), **WB-3** (100% refined wheat flour with 3 g *β*-glucan and stevia extract) **WB-6** (100% refined wheat flour with 6 g *β*-glucan and stevia extract). Values presented as the means of triplicate ± standard deviation. The same-colored columns with different small letters are significantly different (*p* ≤ 0.05).

The antioxidant activity of biscuits treated with stevia and dietary fiber significantly (*p* ≤ 0.05) increased compared to control biscuits which recorded 3.16 ± 0.16 DPPH inhibition (Fig. 4). The WB-6 biscuits showed the highest DPPH inhibition activity (8.63 ± 0.33%) followed by WG-6 biscuits (8.40 ± 0.41%). Evidence from multiple studies indicates that stevia extract exhibits considerable antioxidant activity^40,45^. In the current study, the antioxidant capacity of the stevia extract, as assessed by the DPPH radical scavenging assay, was 73.65%. The antioxidant properties of stevia are not predominantly attributable to stevioside, but rather to other constituents within the extract. High phenolic content (flavonoids, tannins, anthocyanins, and chlorogenic acid) is consistently associated with greater antioxidant activity in stevia extracts^45^.

Furthermore, *β*-glucans confer antioxidant effects chiefly through the direct neutralization of free radicals, such as hydroxyl and superoxide. This activity curtails oxidative stress pathways that can cause biomolecular damage^46^. Also, the antioxidant efficacy of galactomannan, extensively studied through in vitro and in vivo assays^47,48^. It is determined by its molecular structure, degree of branching, molecular weight, and any functional group modifications^47^. Moreover, the antioxidant efficacy of galactomannan can be boosted by chemically modifying, degrading, or conjugating it with other bioactive molecules^47,48^.

#### Enzyme inhibitory activities of α-amylase and α‑glucosidase

As a leading non-communicable disease worldwide, Type 2 diabetes mellitus (T2DM) is a major contributor to the development of cardiovascular diseases, angiopathies, and various metabolic complications^49–51^. The suppression of α-amylase and α-glucosidase activity represents a validated therapeutic approach for glycemic control. This strategy works by interrupting the enzymatic hydrolysis of dietary starch: α-amylase cleaves starch into oligosaccharides and dextrins, while α-glucosidase acts on these products to liberate absorbable glucose. Inhibiting this process retards glucose absorption and thus lowers postprandial blood glucose^52,53^.

A shown in Fig. 4, the WB-6 biscuits showed the highest inhibitory activities against α-amylase and α‑glucosidase by values of 33.87% and 42.53%, respectively. Also, WG-6 biscuits showed enzyme inhibitory activities of 29.56% and 37.625 against α-amylase and α‑glucosidase, respectively. Furthermore, the α-amylase inhibitory activities were recorded as 24.45% and 22.32% for WB-3, and WB-3 biscuits, respectively, while the α‑glucosidase inhibitory activities were recorded as 35.98% and 27.89% for WB-3, and WG-3 biscuits, respectively. The control sample displayed the lowest inhibitory activities of α-amylase and α‑glucosidase by ratios of 5.30% and 8.21%, respectively. Dietary fiber can increase the inhibitory activity against α-amylase and α‑glucosidase enzymes by several mechanisms.

The chemical mechanism for α-amylase inhibition involves the formation of enzyme-fiber complexes, where the binding of fiber alters the enzyme’s active conformation^54^. Also, dietary fiber such as *β*-glucan and galactomannan can act as a physical barrier to enzymatic activity by forming a network that entraps enzymes and substrates^55^. Moreover, the activity of α-amylase and α-glucosidase enzymes can be impeded by inhibitory compounds, including polyphenols and pectin, which are present on the surface of dietary fiber^56^. The incorporation of dietary fibers significantly (*p* ≤ 0.05) increased the inhibitory activity of the prepared biscuits against α-amylase and α‑glucosidase enzymes, indicating their potential as a functional food for diabetes control.

#### Sensory properties of biscuits

**Fig. 5:**
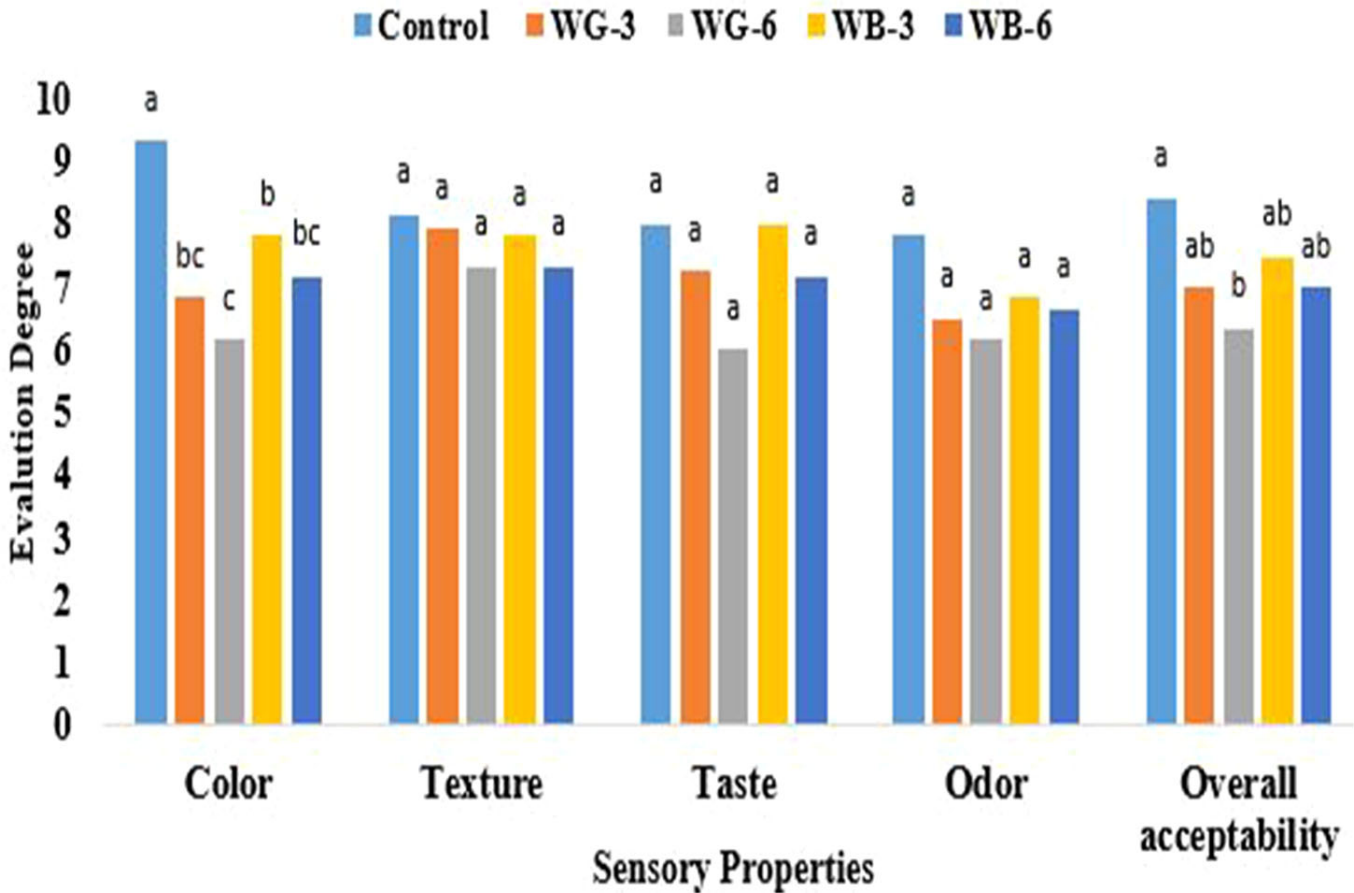
Sensory evaluation of biscuit samples. **Control** (100% refined wheat flour), **WG-3** (100% refined wheat flour with 3 g galactomannan and stevia extract), **WG-6** (100% refined wheat flour with 6 g galactomannan and stevia extract), **WB-3** (100% refined wheat flour with 3 g *β*-glucan and stevia extract) **WB-6** (100% refined wheat flour with 6 g *β*-glucan and stevia extract). Values presented as the means of triplicate ± standard deviation. The same-colored columns with different small letters are significantly different (*p* ≤ 0.05).

Figure 5 showed the sensory evaluation of control, WG-3, WG-6, WB-3, and WB-6 biscuits. For the texture, taste, and odor attributes, there were no significant differences (*p* > 0.05) between the control biscuits and other stevia-treated biscuits. The overall acceptability showed that no significant differences (*p* > 0.05) between control sample and WG-3, WB-3, and WB-6 samples, while significant difference (*p* ≤ 0.05) was observed between control sample and WG-6 sample. The results observed that replacing sugar with stevia extract yielded biscuits with a comparable taste profile. The potential of stevia extract as a successful sugar substitute in food manufacturing has been further supported by the work of ref. ^40^.

The use of stevia extract darkened the biscuits, with a significant color difference (p ≤ 0.05) observed between the control and all stevia-treated samples. The control biscuit was the lightest in color (9.33 ± 0.52), while the WG-6 sample was the darkest (6.17 ± 0.75). The presence of pigments, specifically chlorophylls A and B and carotenoids, is the principal cause of the dark coloration in stevia extract, which often has an undesirable effect on the color of final food products^40^. The development of effective methods to lighten the color of stevia extracts is essential to enhance their suitability for a wider range of food products.

To conclude, results indicated that completely substituting sugar with stevia extract and dietary fibers led to an 11% reduction in biscuit calories. Besides, the substitution of sugar with stevia and dietary fibers resulted in increased antioxidant activity and the inhibition of α-amylase and α-glucosidase enzymes. Among all formulations, the biscuit with stevia extract and 6% *β*-glucan (WB-6) demonstrated superior bioactivity, achieving an antioxidant activity of 8.63% and inhibition rates of 33.87% against α-amylase and 42.53% against α-glucosidase. Sensory analysis indicated no significant (*p* > 0.05) taste difference from the standard product. The incorporation of stevia and dietary fibers enables the industrial-scale production of low-calorie, diabetic-friendly biscuits as a functional food product. Future studies should focus on developing effective methods to lighten the color of stevia extracts is crucial for expanding their use in food products. Moreover, further work is needed to evaluate shelf-life, conduct large-scale consumer trials, and assess the in vivo glycemic response.

## Methods

### Materials

Common oat (*Avena sativa*) was purchased from Agricultural Research Center, Giza, Egypt. Stevia leaves (*Stevia rebaudiana* Bertoni) were obtained from the Sugar Crops Institute, Agriculture Research Center, Giza, Egypt. Fenugreek seeds (*Trigonella foenum*-graecum) cultivar Giza 30 was obtained from Crops Research Institute, Agriculture Research Center, Giza, Egypt. Refined wheat flour was purchased from Alshuruq Mills (Assiut City, Egypt). All chemicals, of analytical grade, were purchased from Sigma Chemical Co. (St. Louis, MO, U.S.A.) via the local supplier, El-Gomhouria Trading Chemicals and Drugs Company in Assiut city.

### Extraction of dietary fibers from oats and fenugreek

*β*-glucan was extracted from whole oats flour using procedure described by Temelli^57^. The whole oats flour (100 g) was suspended in 1000 ml water, pH was adjusted to 7 with sodium carbonate (20%, w/v) and stirred vigorously for 30 min at 55 °C. To remove solids, the mixture was centrifuged for 15 min at 15,000 × *g* at 4 °C. The supernatant was adjusted to pH 4.5 with HCl (2 M) and centrifuged again for 20 min at 21,000 × *g* and 4 °C to separate precipitated proteins. *β*-glucan was precipitated by addition of an equal volume of ethanol (99.9%) to the supernatant slowly with stirring. The precipitate was allowed to settle overnight at 4 °C and was then recovered by centrifugation (10 min, 3300 × *g*). The sample was filtered, washed with 100 ml ethanol (99.9%), air dried to constant weight and then grinded. Light-tan colored powdered gum products were stored at 4 °C till analysis.

Seeds of fenugreek were soaked in water at room temperature overnight. The galactomannan was subsequently extracted from the soaked fenugreek and purified following the procedure outlined by Rashid et al.^58^. The cleaned whole seeds were crushed and then soaked for 24 h at 50 °C in a 5% saline solution acidified to pH 3.0 with acetic acid. The polysaccharides were extracted separately by muslin cloth. Crude gums were purified by the addition of IPA spirit (a blend of 10% isopropanol and 90% ethanol in a ratio of 3:1) with continuous stirring followed by centrifugation at 6000 rpm for 7 min. The white precipitate obtained from fenugreek seed gums was filtered by muslin cloth. The pomace of seeds was again immersed in a 5% salt solution of pH 3.0 until the maximum gum was extracted following the purification procedure. The purified polysaccharides were dried in an oven at 50°C for 24 h. The purified dried polysaccharide was weighed and stored in airtight jars at a cool dry place for further utilization and analysis.

### Preparation and determination of sweetening pawer of stevia leaves extract

The sweetening power of the prepared aqueous stevia leaf extract was evaluated sensorially according Sulaiman et al.^40^. Powdered leaves were steeped in hot water (65°C) at a ratio of 1 g per 35 mL for 3 h. For optimal stevioside extraction, a solvent-to-solid ratio of 1:35 was found to yield the maximum stevioside content. Following centrifugation at 4000 rpm for 15 min and filtration through Whatman No. 4 filter paper, the crude stevioside extract was purified by treatment with calcium hydroxide (0.05 g/g stevia powder). The solution was filtered and the resulting clear extract was then neutralized to a pH of 8.5 using citric acid. The extract was stored at 5–7 °C until use. After preparation of stevia leaves aqueous extract, its sweetening power was sensory evaluated according to Savita et al.^59^ (by a ten-member sensory panel, selected from the Department of Food Science and Technology, Faculty of Agriculture, Al-Azhar University). In the biscuit preparation, 30 g of sugar was replaced on an iso-sweet basis with 27 mL of an aqueous stevia leaf extract, based on the determined equivalence wherein 0.9 mL of extract possesses the sweetening power of 1 g of sucrose (Table 5).

**Table 5:** Formulation of biscuits

Ingredients	Control	WG-3	WG-6	WB-3	WB-6
Wheat flour (gm)	100	100	100	100	100
Sugar (gm)	30	---	---	---	---
Shortening (gm)	20	20	20	20	20
Stevia extract (mL)	---	27	27	27	27
Galactomannan (gm)	---	3	6	---	---
*β*-Glucan (gm)	---	---	---	3	6
Sodium chloride (gm)	1	1	1	1	1
Sodium bicarbonate (gm)	0.5	0.5	0.5	0.5	0.5
Ammonium bicarbonate (gm)	1	1	1	1	1
Baking powder (gm)	0.3	0.3	0.3	0.3	0.3
Water (mL)	16	---	---	---	---

Control (100% refined wheat flour), WG-3 (100% refined wheat flour with 3 g galactomannan and stevia extract), WG-6 (100% refined wheat flour with 6 g galactomannan and stevia extract), WB-3 (100% refined wheat flour with 3 g *β*-glucan and stevia extract) WB-6 (100% refined wheat flour with 6 g *β*-glucan and stevia extract).

### Preparation of biscuits

Five distinct formulations were developed using white flour and other ingredients (Table 5). These were designated as Control (100% refined wheat flour), WG-3 (100% refined wheat flour with 3 g galactomannan and stevia extract), WG-6 (100% refined wheat flour with 6 g galactomannan and stevia extract), WB-3 (100% refined wheat flour with 3 g *β*-glucan and stevia extract), and WB-6 (100% refined wheat flour with 6 g *β*-glucan and stevia extract). Biscuits were prepared according to the method of Manohar and Rao^60^, and sugar was replaced by the stevia extract in all treatments except control sample as presented in Table 5.

### Determination of functional properties of biscuit formulations

The water-holding capacity and solubility were measured in the biscuit formulations as described by Ye et al.^26^ and Singh and Singh^61^, respectively. The oil-holding capacity of the biscuit formulations was assessed following the procedure outlined by Ye et al.^26^.

### Rheological properties of biscuit formulations dough

The influence of adding of stevia extract, galactomannan (3 g, 6 g/ 100 g wheat flour) and *β*-glucan (3 g, 6 g/ 100 g wheat flour) on dough rheological behavior of biscuit formulations was investigated using Mixolab (Chopin, Tripette et Renaud, Paris, France), which provides simultaneous analysis of dough characteristics during the mixing stage under constant temperature, as well as during the controlled thermal cycle of heating and cooling. The amount of flour needed for the analysis was calculated by the Mixolab software using the provided flour moisture and water absorption values. Measurements followed the standard Mixolab Chopin protocol: an initial 8-min hold at 30 °C, heating to 90 °C over 15 min (4 °C/min), a 7-min hold at 90 °C, cooling to 50 °C over 10 min (4 °C/min), and a final 5-min hold at 50 °C. All samples were mixed at a constant speed of 80 rpm, with a fixed dough weight of 75 g and a total analysis duration of 45 min. A standard Mixolab curve is characterized by five distinct phases: (1) initial dough development, (2) protein weakening, (3) starch gelatinization, (4) cooking stability, and (5) starch gel formation during cooling^62^.

### Determination of gross chemical composition of biscuits

The nutritional composition of the biscuits (moisture, protein, crude fiber, crude fat, ash reducing sugar, and non-reducing sugar) was analyzed in triplicate using AOAC^63^ methods. Carbohydrate content was determined by differences. The caloric value was then calculated using the Atwater factors of 9 kcal/g for fat and 4 kcal/g for both protein and carbohydrate, as per Livesey^64^.

### Determination of color properties of biscuits

With the CIELAB color space, the instrumental color values of biscuits were measured according to Hassan et al.^28^ using a manual chromameter (PCE-CSM4, Instruments UK Ltd.). The color values (L* for lightness, a* for redness, and b* for yellowness) were measured at multiple points along the pasta bundle.

### Determination of physical properties of biscuits

Width, thickness, spread ratio (%), and spread factor (%) were measured for a set of six biscuits, with the average values recorded. The spread ratio and spread factor were determined following the method described by Manohar & Rao^65^ as follows:

Spread ratio= Width/ Thickness

Spread factor= (Spread ratio of sample/ Spread ratio of control) *100

### Determination of antioxidant activity

One gram of biscuit samples was combined with 10 mL of chilled ethanol/water solution (80:20, v/v) and agitated at 250 rpm for 2 h at 25 °C. The mixture was then centrifuged at 3000 rpm for 10 min. The combined supernatants were evaporated under reduced pressure (20 mbar) at 50 °C. The radical scavenging activity of the resulting extract was evaluated using the 1,1-diphenyl-2-picrylhydrazyl (DPPH) assay^28^. A volume of 900 µL of DPPH reagent (0.1 mM in 95% methanol) was combined with 100 μL of extract in glass test tubes. Following vigorous mixing, the samples were incubated in darkness at room temperature for 30 min. Absorbance was subsequently measured at 517 nm. The percentage of radical scavenging activity was expressed as scavenging (%) using below equation∶

$$\% {Scavenging}=({Abs}\,{blank}-{Abs}\,{sample})/{Abs}\,{blank}\times 100$$

### The α-amylase and α-glucosidase inhibitory activities

The α-amylase inhibitory activity was assessed using a method adapted from Lu et al.^66^, with slight modifications. In brief, 0.4 mL of α-amylase solution (2 U/mL) was combined with 0.2 mL of polysaccharide solution and pre-incubated at 37 °C for 10 minutes. Subsequently, 0.3 mL of starch solution (5%) was introduced as the substrate, followed by an additional 15-minute incubation. To terminate the reaction, 2 mL of DNS reagent—containing 10 mg/mL 3,5-dinitrosalicylic acid and 120 mg/mL sodium potassium tartrate in 0.4 mol/L sodium hydroxide—was added. The mixture was then heated at 100 °C for 15 min. After cooling to ambient temperature, absorbance was recorded at 540 nm.

The α-glucosidase inhibitory activity was determined according to the method of Xu et al.^67^, with slight modifications. A 50 μL aliquot of polysaccharides solution was combined with 100 μL of α-glucosidase solution (1 U/mL in 0.1 M phosphate buffer, pH 6.9) in a 96-well plate and incubated at 25 °C for 10 min. Subsequently, 50 μL of 5 mM pNPG substrate was added to each well. Following a further 5-min incubation, the absorbance at 405 nm was measured using a microplate reader (Persee M5, Beijing, China), with readings taken both before and after this final incubation period. Acarbose served as positive control. The α-amylase and α-glucosidase inhibitory activities were calculated as follows: % Inhibition activity= [(Abs C - Abs S)/Abs C]*100

Where Abs C is the absorbance of control (100% enzyme activity), and Abs S is the absorbance of tested sample.

### Sensory properties of biscuits

A ten-member sensory panel, selected from the Department of Food Science and Technology, Faculty of Agriculture, Al-Azhar University for their descriptive abilities and sensitivity, assessed the prepared biscuits. The evaluation was based on a 10-point hedonic scale^68^, where panelists rated the color, texture, taste, odor, and overall acceptability of the samples.

### Statistical analysis

A one-way analysis of variance (ANOVA) was performed in IBM SPSS Statistics (Version 22, SPSS Inc., Chicago, USA). Significant differences between groups were determined through Duncan’s multiple difference analysis (*p* ≤ 0.05). All analyses were carried out at triple.

## Data Availability

No datasets were generated or analysed during the current study.
